# Etiologies of endometriosis and model systems: is there a risk of a tunnel vision?

**DOI:** 10.3389/fmed.2025.1675051

**Published:** 2025-10-13

**Authors:** Mary Ann Manavalan, Mona Babtain, Myrthe Weessies, Annemiek Nap, Wouter P. R. Verdurmen, Sebastien Taurin, Mai Sater, Roland Brock

**Affiliations:** ^1^Department of Medical Biochemistry, College of Medicine and Health Sciences, Arabian Gulf University, Manama, Bahrain; ^2^Department of Medical BioSciences, Radboud University Medical Center, Nijmegen, Netherlands; ^3^Department of Obstetrics and Gynecology, Radboud University Medical Center, Nijmegen, Netherlands; ^4^Department of Molecular Medicine, College of Medicine and Health Sciences, Arabian Gulf University, Manama, Bahrain

**Keywords:** endometriosis etiology, fibrosis, organoids, retrograde menstruation, tissue-on-a-chip

## Abstract

Endometriosis is the growth of endometrial-like tissue at non-uterine locations, primarily within the peritoneal cavity. The disease can have diverse presentations with superficial lesions, deep invading lesions and ovarian cysts (endometrioma) as the main subtypes. Immune dysregulation, recurrent inflammatory processes and fibrosis are commonalities of all endometriosis forms. Most theories explaining the etiology of endometriosis take their origin in retrograde menstruation. However, other theories have been proposed, including metaplasia of mesothelial tissue, abnormal proliferation of Müllerian duct embryonic tissue remnants and a stem cell origin. We here argue that there is a lack of attention on whether retrograde menstruation can equally explain the various forms of endometriosis or whether the different endometriosis subtypes differ in etiology. As we show, there is a strong case in favor of several etiologies, as retrograde menstruation alone would require too many assumptions for some clinical appearances of endometriosis. Specific histological and molecular signatures have been associated with the different proposed etiologies. However, these are not part of the standard histopathological characterization of an endometriosis lesion. In addition, current *ex vivo* model systems aim to reconstitute the overall histological structure of a lesion but do not address the potential consequences that different etiologies may have on function and response to therapy. We thus propose to rethink the current diagnostic approach and direct research more specifically toward the cellular and molecular mechanisms underlying the various proposed etiologies, which should then be reflected in *ex vivo* model systems.

## 1 Introduction

Endometriosis is the most frequent gynecological pathology affecting up to 10% of persons assigned female at birth in reproductive age (1). Symptoms vary, ranging from asymptomatic to severe impairment of quality of life, including debilitating pain and infertility. In the same way, the presentation of the disease is highly heterogeneous.

**Figure 1:**
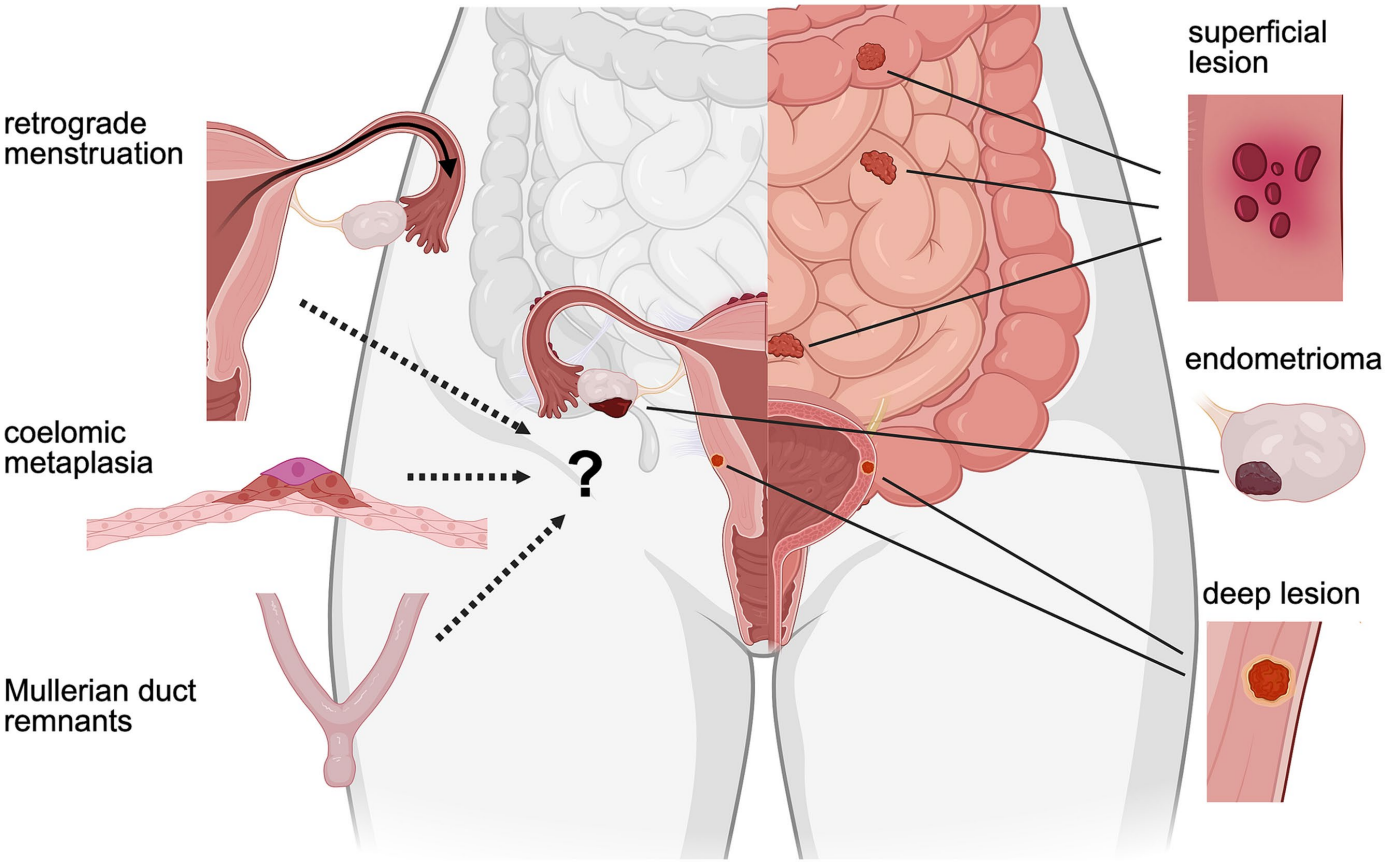
Etiologies and lesion types in endometriosis. Retrograde menstruation, the most widely studied etiology, posits that refluxed endometrial cells during menstruation implant in the peritoneal cavity, forming superficial lesions, deep infiltrating lesions, and endometriomas (ovarian cysts). Coelomic metaplasia suggests that peritoneal mesothelial cells undergo differentiation into endometrial-like tissue, contributing to lesion development. The Müllerian duct hypothesis proposes that embryonic remnants of the Müllerian ducts persist and evolve into endometriotic lesions, particularly deep infiltrating subtypes. Created in BioRender. Brock, R. (2025) https://BioRender.com/4zwz9th.

Endometriosis comprises of growth of tissue with endometrial characteristics in or sometimes outside the peritoneal cavity. Lesions consist of endometrial glandular structures comprising glandular epithelial cells surrounded by stromal cells, similar to the eutopic endometrium. Lesions typically become vascularized, innervated, and infiltrated by immune cells (2). Lesions can be classified based on their histopathology and the anatomical location (Figure 1). Superficial peritoneal lesions are the most common, present on the subserosa soft tissue of the peritoneum (3). Deep infiltrating lesions (DIE) invade over 5 mm into the muscular layers of organs, and are commonly found in the bowel, bladder, or in the rectovaginal septum (4). Ovarian cysts or endometriomas are often filled with blood and tissue (chocolate fluid). These three types of lesions can either exist alone or in combination in the same patient. Overall, there is only a weak correlation between the histological presentation, type of endometriosis, and the severity of symptoms (5–7).

While the majority of the lesions are located within the peritoneal cavity, lesion are also found outside the pelvis (extrapelvic endometriosis), however, such cases are rare with a reported prevalence of (0.04–5.5%) among patients with peritoneal endometriosis. The abdominal wall represents the most prevalent extrapelvic site, even though occurrence at this location is mostly associated with surgical intervention, while other affected regions may include the gastrointestinal tract, urinary system, pulmonary structures, umbilicus, inguinal area, mammary tissue, pelvic nerves, abdominal surgical scars, and exceptionally, cerebral involvement (8). Additionally, adenomyosis, which is a condition characterized by the presence of endometrial tissue in the muscle wall of the uterus, is a frequent comorbidity of endometriosis with a prevalence of 58 to 90%, depending on the diagnostic method and criteria used (9).

Several theories have been proposed to explain the etiology of the disease. The retrograde menstruation theory is the most widely accepted and proposes that endometrial cells and menstrual blood enter the peritoneal cavity through the fallopian tubes during menstruation. The endometrial cells then engraft in one or more locations throughout the peritoneum. This theory was already formulated about 100 years ago (10) and has since then received widespread support through molecular techniques. Endometriotic lesions and eutopic endometrium from the same patients share identical somatic mutations in critical genes (e.g., KRAS, PIK3CA), suggesting a common clonal origin, thereby backing the retrograde menstruation theory (11).

Fresh eutopic endometrium, peritoneal lesions, and surrounding tissue contain numerous SUSD2-positive cells, markers for endometrial mesenchymal stem/stromal cells (eMSC). These cells display angiogenic, perivascular, and immunomodulatory gene expression profiles, indicating their vital role in lesion survival and progression (12). The presence of eMSCs supports the retrograde menstruation theory by suggesting that eMSCs shed during menstruation enter the peritoneal cavity, survive, and contribute to lesion formation. However, to confirm that eMSCs are primarily responsible for peritoneal stromal lesions, further scRNAseq studies on paired menstrual and peritoneal fluid samples are needed (13).

As an alternative to a uterine origin, theories such as coelomic metaplasia, embryonic rest, and stem cell hypotheses have been suggested (14). The concept of coelomic metaplasia proposes that, in response to an unidentified stimulus, mesothelial cells transform into endometrial cells (15). Similarly, the embryonic rest theory implies that cells of Müllerian origin could differentiate into functional endometrium after exposure to an undefined stimulus. The uterus, fallopian tubes, and the upper third of the vagina arise from the Müllerian ducts, which implies that multiple structures within the pelvic cavity share a Müllerian origin (16). Another theory related to stem cells proposes that stem cells from the endometrium could enter the pelvic cavity through retrograde menstruation and subsequently differentiate and give rise to endometriosis lesions (17). Nevertheless, it is important to note that this latter mechanism may be considered a variation of retrograde menstruation. Lastly, it has been hypothesized that endometrium-like cells could emerge from bone marrow stem cells (18, 19).

While retrograde menstruation is a significantly more favored concept than other theories, other factors such as genetic predisposition, environmental factors, and alterations in immune and endocrine functions must also contribute, as retrograde menstruation is more frequent than endometriosis (13). Normally, shed endometrial fragments are cleared by immune surveillance, but in endometriosis, this process is impaired (20). Activated neutrophils and macrophages release cytokines and growth factors that promote angiogenesis, invasion, and the survival of ectopic cells (21). Natural killer (NK) cells, although not consistently reduced in number across studies, are widely reported to exhibit diminished cytotoxicity and limited infiltration into ectopic sites (22, 23). This dysfunction is partly attributed to elevated IL-6 levels in the peritoneal fluid, which suppresses NK cell activity (24). Collectively, these alterations result in defective clearance and a pro-inflammatory, fibrotic peritoneal environment that may drive mesothelial cell transformations, such as epithelial-to-mesenchymal transition (EMT), potentially leading to metaplasia and lesion formation. Once primary lesions have been established, there is also the possibility of metastatic spread within the peritoneal cavity or via the lymphatic or blood system, leading to distant lesions in areas such as the lungs and the brain.

So far, no single (or combination of) precondition(s) has been linked to endometriosis. Thus, endometriosis is most likely a multifactorial disorder and different etiologies could account for the variability in disease presentation and symptoms (13). Endometriosis has been intensely researched *in vitro* using cell lines and primary cell cultures. More recently, 3D disease models have been playing an increasing role for functional studies. These models generally focus on reconstructing the microenvironment of lesion implantation and the cellular composition and histological structure of the lesion. By comparison, the etiology of the formation of the initial lesion or the maturation of the lesion over time have been largely overlooked. Instead, disease models are designed with the premise of retrograde menstruation as the only etiology of endometriosis.

In this review, we summarize the experimental findings supporting the proposed etiologies in relation to the diverse manifestations of endometriosis. We demonstrate that the retrograde menstruation theory is supported by examining critical disease events and functional studies in animals and *ex vivo* models. By comparison, evidence for other etiologies is mainly circumstantial, though this could also stem from the easier access to model systems recapitulating retrograde menstruation. We argue that specific analyses, such as investigating potential monoclonality for lesions deriving from stem cells, should be used more widely. With reasonable additional effort, such investigations could help to scrutinize the potential role of different etiologies. Also, the lack of support from *ex vivo* models for other etiologies than retrograde menstruation may stem from the fact that the relevant events are more difficult to mimic. We thus discuss the challenges and possibilities associated with recapitulating the relevant processes.

## 2 Retrograde menstruation

### 2.1 Occurrence of retrograde menstruation—evidence linking retrograde menstruation to superficial lesions

#### 2.1.1 Endometriosis is linked to a higher propensity for retrograde menstruation

Support for retrograde menstruation was established 40 years ago from a series of seminal publications in humans and baboons. To assess the incidence of retrograde menstruation in persons with and without endometriosis, pelvic fluid from menstruating women was collected during laparoscopy. The pelvic fluid was classified based on color as either straw, pink or bloody with the latter two designations presumed to reflect menstrual bleeding. In the proliferative phase, 61% of women without endometriosis exhibited pink or bloody pelvic fluid compared to 80% of women with endometriosis. In the secretory phase, these numbers increased to 73 and 90% demonstrating an increased incidence for women with endometriosis (25). Notably, most of the non-affected patients also showed retrograde menstruation. The higher incidence of bloody pelvic fluid in women with endometriosis may be explained by retrograde vaginal/uterus peristalsis, which may promote the formation of lesions.

Comparable findings were observed in female baboons. In this study, endometriosis was detected by endometrial glands and stroma, and the peritoneal fluid was categorized as either clear or blood-stained. A 10-fold increase in blood-stained peritoneal fluid was observed during the menstrual phases, compared to other cyclic stages (62% vs. 6%). Moreover, all five baboons with spontaneous endometriosis exhibited recurrent retrograde menstruation in two repeated laparoscopies, compared to only three out of eight unaffected baboons (26).

To ascertain the progressive nature of endometriosis, D’Hooghe and colleagues performed repeated laparoscopies on baboons with spontaneous endometriosis over 32 months (27). The evolution of each endometriotic lesion was followed. In five out of 12 baboons, the total number of lesions, and their American Fertility Society (AFS) scores increased, suggesting that endometriosis progresses in conjunction with menstrual cycles.

#### 2.1.2 The menstrual effluent differs in women with endometriosis

Women with endometriosis exhibited an increased presence of basal layer fragments in the menstrual effluent (28). Due to its regenerative capacity, the basal layer is being considered a source of stem cells for lesion formation (29, 30). In addition, estrogen receptor α (ERα) and progesterone receptors A and B (PR-A/B) were significantly more frequently detected in the epithelium and stroma of the menstrual effluent of the endometriosis group. While these receptors are not specific markers of the basal layer, other studies have shown that their expression pattern is carried across the menstrual cycle. Throughout the proliferative phase, ER and PR are expressed in all endometrial layers, but during the secretory phase their expression declines in the functional layer while being retained in the basal layer (31). Beyond steroid receptors, enzymes involved in intracrine steroid metabolism have also been implicated in endometriosis. For example, P450 aromatase, which is normally absent in healthy endometrium but expressed in stromal cells of endometriotic lesions, contributes to local estrogen production (11). Other enzymes, such as 17β-hydroxysteroid dehydrogenases, sulfatases, and sulfotransferases, have key roles in controlling intracrine estrogen and progesterone levels within lesions (32, 33). Notably, studies analyzing menstrual effluent through transcriptomics, including single cell RNA-sequencing, reveal that stromal and fibroblast populations in women with endometriosis display altered phenotypes compared to controls, potentially supporting local steroid metabolism (34). Overall, these findings suggest that menstrual effluent from women with endometriosis may not only contain more basal endometrial fragments but also reflect an altered hormonal environment driven by receptor expression and abnormal activity of steroidogenic enzymes.

#### 2.1.3 Distribution of lesions supports origin from attached cells

The spatial distribution of endometrial lesions corroborates their origin from disseminated and adhered cells, further supporting the endometrial origin hypothesis. Of 77 people afflicted with endometriosis, 43 exhibited endometriosis lesions solely on the left side, five solely on the right and 29 bilaterally (35) which can be explained by the fact that the peritoneal fluid gets drawn to the upper abdomen preferably via the left side due to a low subdiaphragmatic pressure circulating clock-wise (36).

Moreover, the anatomical positioning of the uterus profoundly impacts the distribution of endometrial lesions. For 182 patients with endometriosis and infertility who underwent laparoscopy, most lesions were located on the ovaries, with the posterior cul-de-sac as the subsequent prevalent location. The exclusive inclusion of infertile women in the study did likely bias the lesion distribution. Among women with an anterior uterus, 40.7% were found to have anterior endometriosis, as opposed to 11.8% in those with a posterior uterus (37). The most plausible explanation for this observation is the external cell deposition within the peritoneal cavity dictated by environmental factors.

#### 2.1.4 Endometrial cells attach to mesothelial cells

Upon entry into the peritoneal cavity, endometrial cells need to adhere to the mesothelium followed by engraftment. To investigate the attachment of endometrial stromal cells to peritoneal mesothelial cells, endometrial stromal cells were isolated from endometrial tissue of several patients obtained during the proliferative phase via pipelle or post-hysterectomy. Additionally, anterior abdominal wall peritoneum was collected from endometriosis-unsuspected women. The endometrial stromal cells showed donor-dependent differences in binding to the peritoneal mesothelial cells. Remarkably, the endometrial stromal cells of a specific patient showed a consistently high/low capacity to bind to peritoneal mesothelial cells of different donors, suggesting that the endometrial donor has a more substantial impact on binding capacity than the peritoneal mesothelial origin (38). Another study also investigated the impact of the menstrual phase of the donor on the attachment of endometrial cells to peritoneal cultures but could not find differences in attachment for proliferative and secretory endometrium fragments (39).

Nap and colleagues addressed the impact of the integrity of the endometrial tissue on endometriosis-like lesion formation. For this purpose, biopsies of cyclic and non-cyclic endometrium along with menstrual effluent were harvested from women without endometriosis and transplanted onto chicken embryo chorioallantoic membrane (CAM). After 24 h, the cells interacted with the CAM mesenchyme. At 48 h, endometrial gland lesions with heterogenous stroma and blood vessels, and after 72 h, organized lesions similar to normal endometrium and endometriotic lesions were visible. Approximately 44% of menstrual effluent samples formed lesions on the CAM, suggesting that endometriosis lesions can arise from endometrium (40).

In endometriosis, the altered expression of cell adhesion molecules (e.g., integrins α4β1/α5β1) is associated with inflammatory processes driven by TNFα, TGFβ, and IL-1, which promote lesion adhesion, survival, and fibrosis (41). For example, in samples obtained from endometrium, menstrual effluent, peritoneal fluid, endometriosis lesions/peritoneum in the early follicular phase (considered the most relevant phase for cell attachment) integrin α4β1 was only detected in cells from endometriosis patients, but not from patients without endometriosis (42). Similarly, integrins present in endometriosis varied from those expressed in eutopic endometrium, with elevated expression of α4β1 and α5β1, suggesting an involvement of fibronectin receptors in endometriotic cell adhesion during menstruation (43).

#### 2.1.5 Retrograde menstruation and superficial lesions—summary

To conclude, retrograde menstruation is sufficient to explain superficial endometriosis due to (i) increased reflux menstruation in both human and baboons with endometriosis, and a progression of endometriosis with the number of menstrual cycles, (ii) attachment of endometrial stromal cells to peritoneal mesothelial cells after 1 h of contact and the endometriosis-specific expression of adhesion molecules, (iii) the localization of endometriosis sites influenced by the anatomy of the peritoneal cavity and the uterine location, and (iv) the capacity of the menstrual effluent to form endometriotic lesions on CAMs.

### 2.2 Evidence linking retrograde menstruation to endometrioma

While superficial lesions affix themselves to the mesothelium without stromal invasion, endometriomata penetrate profoundly into the ovaries. The genesis of these lesions has been explained by the disruption of mesothelial integrity during ovulation, serving as entry point for endometrial cells originating from retrograde menstruation.

To substantiate this hypothesis, women who had undergone a surgical removal of an ovarian cyst received a series of ultrasound scans post-surgery. When a hemorrhagic corpus luteum cyst was identified, weekly transvaginal ultrasounds ensued, continuing until resorption or conversion into an endometriosis cyst was observed. If the conversion was found, another ultrasound was conducted after 2 months to confirm the diagnosis of endometrioma. Out of the 109 women who completed the study, only 13 exhibited a hemorrhagic corpus luteum, with 11 of these cases undergoing transition to endometrioma. However, 14 women were diagnosed with endometrioma without a prior hemorrhagic cyst. The researchers could not exclude the potential omission of the hemorrhagic corpus luteum (44).

However, based on histological evidence, in particular the presence of ovarian cortex around the cysts, different mechanisms for formation of endometriomata have been proposed. Invagination of the ovarian cortex can also occur if endometriotic lesions are attached to ligaments close to the ovaries and this attachment blocks the egress of fluid from the lesion (45). This latter mechanism could either result from retrograde menstruation or alternatively from coelomic metaplasia (see below).

### 2.3 Evidence linking retrograde menstruation to deep infiltrating endometriosis

Deep infiltrating endometriosis (DIE) is defined by lesions extending over 5 mm beneath the mesothelial surface (46). Evidence linking this form of endometriosis to retrograde menstruation remains limited. Already in 1927, Sampson studied the uterine anatomy and blood supply through liquid gelatin-perfused veins and arteries followed by hardening with paraffin and postulated his initial and largely forgotten theory that the menstrual-endometrium interface could impair the venous endothelium, thereby permitting the entry of endometrial tissue into the bloodstream. Mucosa of the menstruating uterus was found within the veins, yet the possibility of these observations being artifacts could not be excluded. In the case of a uterus containing an embolus-like endometrial tissue lesion, its histological connection with vein contents and linings rendered it highly improbable that the endometrial tissue presence was an artifact. Remarkably, this uterus belonged to a patient afflicted with severe endometriosis (47). Only subsequently, Sampson proposed retrograde menstruation through the fallopian tubes. In 1940, Sampson expanded his theory to show that after implantation, these endometriotic lesions penetrate the underlying structures and spread in a metastatic manner (48).

The conclusive determination of whether vascular reflux may cause DIE necessitates careful exploration of the anatomy of blood circulation, particularly the proximity of uterine veins to common DIE locations. Furthermore, the fragments would be required to leave the circulation, which should predominantly occur within the peritoneal cavity rather than externally, given the higher frequency of DIE compared to extra-pelvic manifestations.

Evers et al. studied the ability of endometriosis cells to infiltrate the basal membrane. A total of 16 endometriosis biopsies were taken, four biopsies of each endometriosis type as per the AFS classification. The study mainly questioned whether the distribution of collagen IV and the integrity of the basal membrane could indicate disease severity. Collagen staining patterns were uniform, and the basal membrane remained intact throughout all cases, thus refuting the infiltrative capabilities of endometriotic lesions (49). Such findings indicate that deep endometriosis lesions do not originate from local tissue. On the other hand, Solaris et al. analyzed 17 biopsies of deep infiltrating lesions and identified a role of collective cell migration (CCM) in the movement of endometrial glands from the surface to deep muscle tissue. In CCM, groups of cells maintain their cell–cell junctions as they collectively move to invade the surrounding tissue (50).

Interestingly, as for superficial lesions, there was a preference of peritoneal and abdominal DIE lesions for the left side. Again, these findings suggest a correlation between the flow of peritoneal fluid and the development of DIE lesions supporting a role of retrograde flow in the pathogenesis of DIE (51).

In conclusion, retrograde infiltration can explain DIE. However, the route along which the fragments enter the peritoneal cavity is still disputed.

### 2.4 Evidence linking retrograde menstruation and abdominal wall endometriosis

Abdominal wall endometriosis is a rare form of endometriosis, defined by the presence of endometrial tissue within or along the abdominal wall and superficial to the peritoneum (52). It has been hypothesized that endometrial fragments present in the peritoneal cavity are deposited in the abdominal wall via surgical instruments (53).

Support for this hypothesis was derived from an investigation conducted on 82 women exhibiting abdominal wall endometriosis, which was referred to as cutaneous endometriosis in this study. Fifty six lesions were located at the surgical scar, while 26 lesions developed spontaneously. Among the 26 patients with spontaneous lesions, 21 women had not undergone any prior surgery, while for the remaining five, the prior surgery was not in the vicinity of the lesions. Unfortunately, the documentation of concurrent pelvic endometriosis for the majority of the 82 women is limited, rendering it inconclusive as to whether endometrial fragments were transported as a consequence of retrograde menstruation or injury to the endometrium, or if the lesions emerged independently of surgical intervention (54). Even though retrograde menstruation is the most likely route for endometrial fragments to enter the peritoneal cavity, it is remarkable that in a study of women with cesarean scar endometriosis, none had a previous history of endometriosis, which suggests that the development of abdominal endometriosis could be a consequence of the surgical intervention (55). A third possibility, which was first introduced by Halban in 1924, involves lymphatic or hematogenous dissemination. This mechanism may also explain the occurrence of spontaneous abdominal wall lesions in women without any prior history of surgery or pelvic disease. In conclusion, while retrograde menstruation likely contributes to some cases, the evidence indicates that it does not fully explain all instances of abdominal wall endometriosis, especially those spontaneous lesions that arise in the absence of pelvic disease or prior surgical procedures (56).

## 3 Coelomic metaplasia

In contrast to retrograde menstruation, the coelomic metaplasia theory postulates the formation of endometriotic lesions as a result of the differentiation of peritoneal epithelium into endometrial or endometrial-like tissue within the peritoneal cavity. This theory primarily arose to account for the infrequent occurrences of endometriosis in males and individuals with Mayer-Rokitansky-Küster-Hauser (MRKH) syndrome, characterized by an underdeveloped uterus and vagina. However, a recent study has shown a significantly higher prevalence of endometriosis in MRKH patients with functional endometrium (32.0%) compared to those without (1.5%), suggesting that retrograde menstruation could be the primary driver of endometriosis in this population, challenging the coelomic metaplasia theory as the dominant etiology (57). Furthermore, the presence of endometriosis in pulmonary tissue, which possesses a mesothelial component, may be explained by the coelomic metaplasia theory (15). Coelomic metaplasia is assumed to be initiated by several stimuli such as endometrial stroma-derived growth hormones and cytokines (58).

### 3.1 Evidence linking superficial peritoneal endometriosis to coelomic metaplasia

Evidence has been presented that the mesothelium can undergo metaplastic transformations, ultimately giving rise to structures resembling endometrium. Hematoxylin and eosin (H&E) staining of blueish-colored ovarian surface endometriosis lesions obtained through laparoscopies of 26 women showed endometrial gland-like tissue beneath the epithelium. The sunken glands displayed three distinct types of epithelia with varying densities of microvilli. Furthermore, the endometriosis lesions were positive for cytokeratin (epithelium) and vimentin (stroma), consistent with standard endometrial staining. The staining of infolded mesothelial cells paralleled that of endometrial glands and endometriosis tissue, suggesting a transition from mesothelium to endometriosis through metaplasia. The authors proposed that the stimulus for the metaplasia lies within the retrograde menstrual products, rendering retrograde menstruation the leading cause of the origin of endometriosis, though not at a cellular level (59).

Additionally, microscopic analysis of the superficial peritoneal lesions showed the presence of columnar mesothelial cells with peritoneal infoldings in pelvic peritoneal endometriosis with immunoreactivity similar to normal endometrial glands, suggesting a transformation of peritoneal mesothelium into endometrial-like tissue. The presence of ciliated cells at the edges of these infoldings, that resemble endometrial glands further aligns with the theory that pelvic endometriosis may arise through metaplasia of the coelomic-derived peritoneal tissue (60).

In further support of metaplasia, ovarian surface epithelium and endometrial stromal cells of patients without endometriosis were cultured on type 1 collagen and subsequently exposed to 17β-estradiol at a concentration 10 times higher than the one found in the peritoneum of endometriosis patients, for 1 week either as single cell types or co-cultures. With the addition of estradiol, the cultures of ovarian surface epithelium formed a ring-shaped cell arrangement with luminal architecture, Alcian blue and cytokeratin-positive lumen surfaces but negative for epithelial membrane antigen. Similarly, a lumen materialized in glandular structures when estradiol was present in co-cultures of ovarian surface epithelium and endometrial stromal cells, in contrast to the estradiol-deprived group. In response to estradiol, endometrial stromal cells alone also developed luminal glandular structures that were absent in the control group. Thus under the influence of estradiol, ovarian epithelial cells alone form luminal structures, indicating metaplastic development of endometriosis involving ovarian surface epithelium and endometrial stromal cells, with estradiol as the stimulant (59). It should be noted that the ovarian epithelial cells were used as a model for the peritoneal mesothelium, thus explaining endometriosis in the broader sense. Two aspects should be noted: first, the use of very high estradiol concentrations, and second, they assumed endometrial stromal cells to be a major stimulant for metaplastic transformation, whose presence can only be explained through retrograde menstruation theory.

### 3.2 Evidence linking coelomic metaplasia and endometriomata

Evidence also suggests that endometriomata might form locally within the ovary, possibly through metaplastic transformation. In one study, researchers examined 110 endometriomata and 30 benign ovaries containing epithelial inclusions not related to endometriosis. They used immunohistochemistry to assess CD10, which identifies endometrial stromal cells, and aromatase, an enzyme involved in estrogen production (mentioned above), as aromatase expression supports the idea that these lesions are hormonally active. CD10 was expressed more strongly in the endometriotic lesions than in the benign ovarian lesions. Interestingly, lesions located deeper in the ovarian cortex showed higher expression of CD10 than those on the ovarian surface. This could indicate that stromal-like differentiation is more prominent in the deeper lesions, possibly due to local transformation of ovarian tissue into endometrial-like cells. That said, it is still possible that these lesions began through retrograde menstruation, with metaplastic change and stromal differentiation occurring later as the lesion continues to develop. However, it cannot be ruled out that endometriosis first developed from retrograde menstruation, followed by differentiation, which then led to the observed disparity in CD10 expression (61). This study also provided evidence on the morphological transition of ovarian surface epithelium and stroma to initial endometriosis in support of the metaplasia hypothesis. In biopsies of the walls of 814 endometriomata after cyst drainage, instances of metaplasia to endometrial tissue occurred in the invaginated mesothelium covering the ovaries. Furthermore, the endometriotic tissue was in continuum with the invaginated mesothelium (62).

### 3.3 Evidence linking coelomic metaplasia to abdominal wall endometriosis

The association between coelomic metaplasia and abdominal wall endometriosis is predominantly established through case studies. A cyst from a 31-year-old patient with abdominal endometriosis showed strong staining for αSMA in the periglandular stroma which the authors postulated as indicative of metaplasia. However, the authors failed to delineate the rationale underpinning this supposition of metaplasia, or to delineate the implications of an endometriosis development devoid of metaplastic origins (63). Furthermore, Yan et al. also demonstrated the presence of mesothelial cells in ovarian endometriosis and DIE, which can undergo mesothelial-to-myofibroblast transition (MMT) on exposure to activated platelets (64), consistent with the presence of peritoneal mesothelial cells within the endometriotic lesions, supporting the coelomic metaplasia theory during the formation of endometriotic lesions.

## 4 Origin from embryonic remnants

The embryonic remnant hypothesis posits that errant migration and differentiation processes occurring during the organogenesis of the female reproductive tract lead to the dispersion of Müllerian duct remnants across the posterior pelvic floor. These remnants consequently transform into endometriotic tissue. Research supports the presence of Müllerian remnants in the cul-de-sac region, a deep structure within the pelvic cavity (65).

### 4.1 Evidence linking embryonic remnants to superficial peritoneal endometriosis

Sections of the pelvic organs of 36 female fetuses at varying gestational ages and presenting no discernible abnormalities were stained for CA125 and the estrogen receptor-α. Both antigens are well-defined markers for the female reproductive tract. In four of the fetuses, CA125 and estrogen receptor-α-positive structures similar to primitive endometrium were identified outside of the uterine cavity, with locations including the rectovaginal septum near the Pouch of Douglas, within mesenchymal tissue adjacent to the uterus’ posterior wall, and along the muscularis propria of the rectal tube (66). Superficial endometriosis lesions are frequently found in these anatomical regions, indicating a link to Müllerian remnants in forming superficial peritoneal endometriosis.

### 4.2 Evidence linking embryonic remnants to deep infiltrating endometriotic nodules

Donnez and colleagues compared the histological and morphological characteristics of endometriotic nodules from the rectovaginal septum with those of peritoneal lesions. The peritoneal lesions demonstrated two distinct types: superficially situated red lesions with higher activity as identified by the presence of stromal glandular epithelium exhibiting proliferative or progestogen-unresponsive characteristics and deeper black lesions with lower activity. Histological sections were stained with Gomori’s trichrome staining alongside immunohistochemistry for cytokeratin, vimentin, estrogen receptor, and progesterone receptor. For the rectovaginal endometriosis, the presence of epithelial features other than endometrium supported a coelomic epithelial origin. The rectovaginal lesions demonstrated histological features of adenomyosis and thus likely originated from Müllerian remnants. This study also showed that different endometriosis nodules react differently to hormonal treatment. Peritoneal endometriosis lesions showed reduced mitotic activity and incidence of active endometriosis after GnRHa (gonadotrophin-releasing hormone agonist) therapy compared to Lynestrenol (synthetic progestin) therapy. In contrast, Lynestrenol therapy reduced vascularization in rectovaginal septum nodules more effectively. This led the authors to suggest that peritoneal and rectovaginal lesions were different diseases and had distinct pathophysiology (67).

In a second, similar study, biopsies of 52 typical black peritoneal lesions and 68 rectovaginal nodules were taken and analyzed using the same markers as in the first study. Reduced co-expression of vimentin and cytokeratin in the rectovaginal tissue below the vaginal mucosa indicated a close relationship of the tissue with a mesodermal Müllerian origin (68).

## 5 Stem cell theory

Finally, endometriosis has been linked to stem cells, a concept that could explain the monoclonal origin of endometriomas (69). There are two distinct hypotheses which either postulate (i) an origin from hematopoietic or mesenchymal stem cells arising from the bone marrow, or (ii) an origin of stem cells from endometrial tissue itself, which may be considered a variant of the retrograde menstruation model in which endometrial stem cells give rise to ectopic lesions.

### 5.1 Evidence linking the stem cell theory to superficial peritoneal endometriosis

A notable study investigated the methylation pattern of the X-chromosome in superficial peritoneal endometriosis specimens. In female somatic cells, one X-chromosome is randomly inactivated. A clonality analysis for endometriosis biopsies of 17 patients indicated that one endometriosis gland originates from one precursor cell, with its proliferative capacity implicating a stem cell origin. Additional findings in the study disclosed a multicellular etiology of different endometriosis lesions, leading to the conclusion that superficial peritoneal endometriosis does not arise from a single stem cell, but rather, multiple stem cells might contribute to its genesis (70). Entry of stem cells into the peritoneal cavity by retrograde menstruation constitutes a plausible route for their ectopic localization, especially considering the stronger shedding of cells from the basal layer observed in endometriosis patients.

### 5.2 Evidence linking the stem cell theory to endometriomata

Monoclonality was also demonstrated for endometriomata. Fourteen endometriosis cysts on the ovaries were removed from 10 women, followed by careful removal of epithelial cells from the inner surface and DNA isolation. Around 70% of cells had a monoclonal origin while the rest were most likely due to incomplete cell separation. However, it was not examined whether the stromal cells also had a monoclonal origin (71).

A separate study investigating epithelial cells of 40 endometriosis lesions from 20 patients obtained evidence for monoclonal origins in 18 out of the 20 patients, but stromal cell monoclonality was once more not addressed (72). Endometriomata were present in 17 of the 20 patients, while the other three exhibited lesions on the peritoneal surface.

### 5.3 Evidence linking the stem cell theory to deeply infiltrating endometriosis

Whole genome sequencing was applied to 24 complete endometriosis lesions comprising of both stroma and epithelium. Five of these were deep infiltrating endometriosis lesions, alongside one ovarian endometriotic cyst. Stroma and epithelium were separated before DNA isolation. Nineteen mutations were discovered, all exclusive to the epithelial component. Two patients exhibited mutational patterns indicative of multiclonality, while the others implied monoclonality. The data suggested that the epithelial stem cells reach the endometriosis site first, and the stromal cells follow. If stroma and epithelium developed out of the same cell, stroma should have contained the same mutations as the epithelium. The authors posit that the stromal component of endometriosis lesions originates from various (stem) cells, but no justification for this conclusion is provided (73).

### 5.4 Evidence linking hematopoietic stem cells to endometriosis

In four women who underwent allogeneic bone marrow transplantation from single-antigen mismatch-related donors possessing diverse HLA types, immunohistochemistry and RT-qPCR of endometrium revealed the presence of endometrial epithelial cells and stromal cells of donor origin (18). The majority of glands were composed of a single cell type (either donor or recipient), while some glands comprised of both. The percentage of bone marrow-derived cells was higher in the stroma compared to the glandular epithelium. The endometrium contained over 50% donor-derived cells in one patient, while the remaining patients had fractions of 0.3, 4, and 11% donor-derived cells. Consequently, this study demonstrated that bone marrow-derived cells can differentiate into human uterine endometrium. Although this finding does not specifically prove the potential for stem cells to cause endometriosis, it provides evidence of stem cells within the circulatory system that can differentiate into endometrial cells.

## 6 The case for different etiologies

Retrograde menstruation readily explains the presence of endometrial-like tissue outside the uterine cavity; however, further conditions are needed, given that retrograde menstruation is also widespread among people without endometriosis. For endometriomata as well, an association of retrograde menstruation with lesion formation has been proposed. The endometrium-derived stem cell theory is a variation of the core concept. This theory aligns with the observation of a greater tendency for basal layer shedding in women with endometriosis.

Nonetheless, histological evidence for metaplasia and the few documented cases of endometriosis in MRKH patients bolster the notion that peritoneal mesothelium may transform into endometriotic lesions. However, as a cautionary note, in most of the case reports of MRKH patients with endometriosis, it was not ruled out/documented that there was no rudimentary uterus and/or functional endometrium, and not in all cases endometriosis was confirmed histologically (74). Additionally, the increased prevalence of endometriosis among MRKH patients with functional endometrium compared to MRKH patients without functional endometrium strongly supports retrograde menstruation as the key etiology of endometriosis (57). Retrograde menstruation could deliver a stimulus that induces metaplasia.

For deep infiltrating endometriosis, invasion from the surface has been proposed next to vascular damage in the uterine mucosa, permitting entry of endometrial cells into circulation, a concept that had already been proposed by Sampson (47). A detailed investigation of the locations of deeply infiltrating endometriosis in relation to vasculature is needed to evaluate further this hypothesis, which could concurrently explain the etiology of extrapelvic endometriosis.

For abdominal wall endometriosis, there is a correlation with abdominal wall surgery; the origin of the cells causing lesions is still likely the endometrium. Such lesions may result from cells residing in the peritoneal fluid during surgery.

It has been demonstrated that rectovaginal lesions of Müllerian origin exhibited differential responses to GnRHA treatment compared to superficial lesions (62). Consequently, devoting greater attention to the underlying etiology might enhance therapeutic outcomes and facilitate the development of novel, more effective, and etiology-specific treatment modalities.

In our perspective, there exists robust evidence for disparate etiological origins. Notably, distinct etiologies, encompassing coelomic metaplasia and retrograde menstruation, may cause histologically very similar lesions at similar loci. Support for these etiologies could be provided through thorough histological examinations, immunohistochemical analyses, and (epi)genetic evaluations, which fall outside the customary diagnostic procedures for endometriosis that primarily focus on ascertaining the benign nature of endometriotic lesions.

## 7 Current *in vitro* and *ex vivo* models to study endometriosis

*In vitro and ex vivo* models are crucial for understanding the cellular and molecular processes in endometriosis. With *in vitro* models we refer to models employing cell lines whereas *ex vivo* models relate to the tissues taken from a patient and maintained in culture.

### 7.1 Two-dimensional model systems

**Figure 2:**
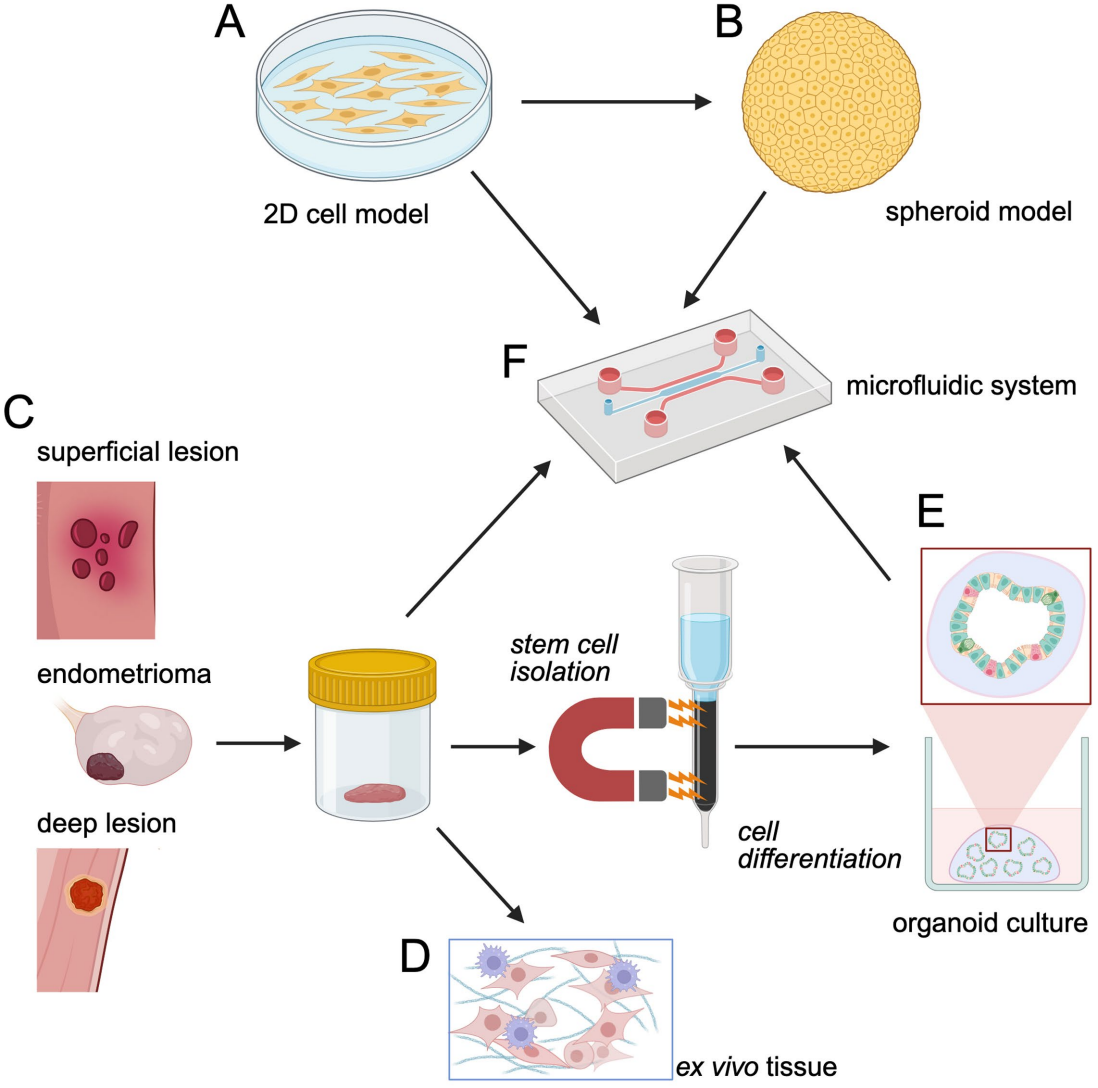
Models of endometriosis. **(A)** 2D tissue cultures of immortalized or primary cells are the most fundamental and easy to handle model from which **(B)** 3D spheroid models can be generated. **(C)** By comparison organoids are obtained through differentiation of stem cells isolated from patient biopsies (either endometrial or endometriosis-derived) in the presence of growth factors in a 3D matrix. **(D)** Alternatively, patient-derived material can be cut into thin tissue slices for direct use in *ex vivo* tissue culture. **(E)** Stem cell-derived organoid cultures recapitulate lesion heterogeneity and cell differentiation. **(F)** All types of models can be implemented into microfluidic devices for tight control of the physiological environment and co-culture of cells such as of spheroids/organoids embedded into a stromal matrix. Created in BioRender. Brock, R. (2025) https://BioRender.com/i3p6hjg.

Two-dimensional (2D) cellular tissue culture models are the most fundamental system to analyse cellular behavior. Since epithelial and stromal cells are key components in endometriosis lesions, there is a justification for 2D tissue cultures to recapitulate at least the epithelial aspect of the lesions. Several endometriosis epithelial and stromal cell lines have been established and are commercially available (75–77). Compared to primary patient-derived cells, the advantages of using these cell lines are easy handling and immortalization. Endometriosis epithelial cell lines that express cytokeratin and N-cadherin have been immortalized and exhibit invasive behavior, as seen *in vivo*. Similarly, endometriosis stromal cell lines that are cytokeratin-negative and vimentin-positive have been established and used to study epithelial-stromal interactions (78) (Figure 2A). The commercially available endometriosis epithelial cell line 12Z has been widely studied and recapitulates many *in vivo* epigenetic changes, including specific DNA methylation patterns and histone modifications, that can affect cell behavior such as cell proliferation, cell cycle regulation and apoptosis (77, 79, 80). This aspect is especially relevant since significant changes in epigenetic regulators for differential chromatin organization and DNA methylation have been observed in endometriosis patients, adding to growing evidence on the role of epigenetics in the pathophysiology of endometriosis (81).

One way to study cell–cell communication is by using transwell systems. In these systems, cells are separated by a semi-permeable membrane and can communicate by paracrine signaling. Woo et al. used this assay to demonstrate increased macrophage migration toward endometriotic epithelial cells (11Z and 12Z) compared to healthy endometrial epithelial cells, mediated by monocyte-chemoattractant protein 1 (MCP-1). Endometriotic epithelial cells also secrete IL-13 and IL-4, initiating crosstalk that mediates M2 polarization of macrophages and increased IL-6 production, which in turn enhances the migration capability of endometriotic epithelial cells (82).

Cells, whether primary patient-derived cells or established cell lines, exhibit different morphology and organization when cultured on a solid support, compared to *in vivo* (83). These changes in organization lead to disturbances in cell signaling, affecting their ability to interact with the external environment (84–87). Culturing cells on an extracellular matrix provides an effective solution to overcome this challenge. It has been shown that culturing 12Z cells on different types of extracellular matrix alters the differentiation and invasion capability of the cells (88). It is also important to note that cells growing in a 2D system typically receive a continuous oxygen supply, whereas endometriotic lesions usually exist in a hypoxic environment. Also in 2D systems, researchers should thus aim to understand the effect of hypoxia on the pathogenesis of endometriosis and accurately mimic this environmental aspect (89).

The established endometriosis cell lines were derived from individual patient lesions, with their unique epigenetic profiles. Moreover, primary cells will undergo further epigenetic changes during immortalization (90). Ideally, primary patient-derived cells should be used to study the highly diverse presentations of endometriosis as they retain the epigenetic profiles of cells in the original tissue, providing a more accurate and comprehensive understanding of endometriosis-related cellular processes. However, without understanding whether and to which extent (epi)genetic differences also translate into functional differences, no recommendations can be made on how many samples to include to obtain a representative coverage of cell behaviors. Additionally, the limited number of epithelial cells that can be harvested from the lesions along with the difficulties in maintaining their viability, pose significant hurdles in their effectiveness as a model to study endometriosis (2).

Apart from endometriotic cell lines, patient peritoneal mesothelial cells have been studied to understand the mechanism of how endometrial cells attach to the peritoneal surface. An intact mesothelium has been shown to prevent the adhesion and invasion of endometrial tissue in menstrual effluent (91, 92). Multiple studies have reported differential expression of adhesion factors in the peritoneum of women with endometriosis. Changes in cell phenotype, like loss of tight junctions, can provide sites for ectopic endometrial cell attachment and establish endometriosis lesions. Additionally, epithelial-to-mesothelial transition of the peritoneal mesothelium, altered metabolism, and lowering of peritoneal immune scavenging promote lesion development and highlight the role of peritoneum in the development and persistence of endometriosis (20).

### 7.2 Endometrial spheroid models

While 2D systems may help to understand the functional characteristics of endometrioid epithelia, they fail to reproduce the three-dimensional glandular epithelial structures embedded into a stroma rich in immune cells. Therefore, three-dimensional cell culture models are increasingly used to study cellular processes in endometriosis. Spheroids are the most basic type of three-dimensional models in which cells are grown without a solid support. The first spheroid model of endometriosis was developed using the endometrial epithelial cell lines 16 and 12Z (83). Under non-adherent conditions, both cell lines self-organized into smooth, symmetrical spheroid structures within 24 h (Figure 2B). The histology and gene expression patterns of the endometrial spheroids were comparable to those of *in vivo* endometriotic lesions. Song et al. developed heterogeneous endometriotic spheroids comprised of endometrial stromal cells in the core surrounded by immortalized epithelial cells (12Z). The ability of the spheroids to invade the peritoneum was studied by seeding the spheroids on a matrigel-mesothelial layer. The spheroids migrated through the matrigel matrix to penetrate the mesothelial cell layer. It was interesting to note that stromal cells were always at the leading edge during the migration and first to establish contact with the mesothelial cell layer (93).

### 7.3 Endometrial organoids

The development of endometrial organoids from primary cells of human and murine endometrium was a breakthrough in three-dimensional *in vitro* models of endometriosis (Figure 2E) (94, 95). In contrast to spheroids, organoids originate from stem cells. Through proper recapitulation of differentiation, organoids can more closely mimic the multicellular composition and structure of the physiological situation such as a glandular structure with a hollow lumen and apico-basal polarity, and release of secretions (96). Moreover, as much as primary epithelial cells, organoids enable the capture of patient-specific characteristics. Organoids were cultured from human adult stem cells obtained from healthy endometrial biopsy samples in a conditioned medium containing multiple growth factors, namely fibroblast growth factor 10 (FGF10), EGF, Wingless and Wnt signaling activator R-spondin 1 (RSP1), Noggin and the TGF-β antagonist A83-01 (94, 95). RNA sequencing showed that endometrial organoids were more genetically aligned with endometrial epithelium than stromal cells. Thus, endometrial organoids closely mimic the eutopic endometrial physiology and can be used to study cellular re-organization, cell–cell interactions, and changes in the extracellular matrix. An interesting future line of research would be to ask what drives these organoids into an endometriosis phenotype.

The initial endometrial organoid models only studied organoids generated from patient-derived endometrial epithelial cells as mentioned above. However, more recent models combined primary endometrial stromal cells and endometrial epithelial organoids in a synthetic extracellular matrix, integrating the two key cellular components of endometrial lesions (97). This system was used to recapitulate cellular changes during the menstrual cycle and to serve as a model for investigating the transition to an inflammatory phenotype in endometriosis. Wiwatpanit et al. hypothesized that the stromal cells could provide a scaffold or support for the epithelial cells as in the native tissue. They developed a scaffold-free endometrial organoid model by co-culturing endometrial epithelial cells and stromal cells isolated from uterine tissue of pre-menopausal women to study polycystic ovary syndrome (98). The organoids exhibited distinct self-organization, with the stromal cells migrating to the center of the organoid and the polarized epithelial cells forming the outer surface of the organoid. The organoids expressed functional and cell-type specific markers, including androgen, estrogen, and progesterone receptors (98). This morphology was similar to spheroids obtained from co-culturing endometriotic epithelial (12Z) and stromal cell lines (93). However, concerning endometriosis research, it is important to note that while these organoids and spheroids have a polarized epithelial outer layer, the center of the organoid is made up of stromal cells, which does not accurately represent the lesion *in vivo*. Endometrial organoid models are advancing, with recent models combining endometrial organoids and stromal cells within different synthetic extracellular matrices. To address the variability across the different organoid models described above, researchers aim to harmonize the generation of organoids to ensure comparability and reproducibility across the models and enhance translational efficiency for therapeutic development (99).

Since the organoids mentioned above were all derived from eutopic endometrial cells, the question arises: what do these models teach us about endometriosis? The organoids teach us about the self-organizing capacity of endometrial epithelial stem cells into glandular structures that reproduce histological and molecular features of endometriotic lesions while providing insight into their responsiveness to ovarian hormones. However, studies that investigate matrix-invading behavior in general, without addressing factors specific to the engraftment of endometrial cells entering the peritoneal cavity by retrograde menstruation, fail to penetrate to the core of the problem; namely, why given the high prevalence of retrograde menstruation, endometriosis is still relatively rare.

### 7.4 Endometriosis-derived organoids

Whereas at least according to the retrograde menstruation theory, endometriotic lesions are derived from endometrium, they have undergone environment-dependent changes in gene expression profiles and epigenetic modifications (100). Thus, endometriosis-derived model systems should behave differently from endometrial model systems, and in particular, comparative studies should be instructive. Boretto et al. performed a comprehensive analysis of organoids derived from endometrium of healthy controls, matched pairs of eutopic and ectopic endometrium, further ectopic endometrium samples and endometrial (pre-)malignancies (101). Principal component analysis showed that organoids derived from healthy and patient eutopic endometrium clustered together, suggesting similarity in gene expression, while the endometriosis-derived organoids showed more variation. The ectopic endometrium-derived organoids had distinct differentially expressed genes, especially increased TGF-β signaling marked by downregulation of *BAMBI* and upregulation of *GDF11* (101). Altered TGF-β signaling in these organoids may modulate their hormonal responses, potentially accounting for progesterone resistance and heightened estrogen sensitivity, both of which are well-documented features of endometriosis (102, 103).

These endometriosis-derived organoids possessed a thicker lumen-bordering cell layer than those derived from patient-derived eutopic endometrium and endometrium of healthy controls. Additionally, endometriosis-derived organoids demonstrated a capacity for luminal invasion, consistent with primary endometriotic lesions. RNA-sequencing analysis of endometriosis-derived organoids exhibited differential gene expression compared to both healthy and patient endometrium-derived organoids, with altered levels of genes related to ECM-receptor interaction (*COL3A1*, *FN1*), PI3K-AKT signaling (*IGF1*, *ITGB8*), WNT signaling (*CTNNA2*, *WNT11*), hormonal response (*PGR*, *HSD11B1*), Hippo signaling (*CTGF*), adhesion/invasion (*MMP2*, *SNAI2*), and developmental regulation (*HOXD8*, *HOXA9*). These findings suggest that endometriosis-derived organoids could respond differently to hormones due to altered expression of genes like *PGR*, *ESR1*, *HSD11B1*, *LIFR*, and *PRLR* alongside pathways (PI3K-AKT, WNT) that amplify the hormonal effects, making it a valuable model to study endometriosis (101).

Structurally, transmission electron microscopy revealed the presence of stratified epithelium in organoids derived from endometriotic lesions, which was not observed in organoids generated from either patient eutopic endometrium or healthy endometrial tissue. Furthermore, injection of the endometriosis-derived organoids into the peritoneal cavity of mice led to the development of lesions that expressed specific endometriosis markers (101). These results establish organoids as an effective model for studying endometriosis that recapitulates the structure of the lesion and could be used to study patient-specific drug responses. As a caveat of this study, neither patient nor control endometrium-derived organoids were tested, thus it is not clear whether lesion development was disease-specific. These experiments were conducted in NOD-SCID mice, thus a model lacking the lymphocyte compartment. More recently, Zhang and colleagues developed endometriotic organoids from endometrioma lesions expressing epithelilal markers and estrogen/progesterone hormone receptors (104). This model offers a promising tool for studying patient-specific drug responses and disease mechanisms.

Organoids from superficial lesions and endometriomata have already been established (101, 104) (Figure 2C). The generation of organoids from deep-infiltrating lesions should enable comparative studies to understand the mechanistic differences between the different types of lesions and give insights into endometriosis pathophysiology. When utilizing these models, it should be noted, however, that it is predicated on retrograde menstruation and stem cell theory as the underlying etiology of the disease.

### 7.5 Endometrial tissue explants

The use of tissue explants or slices from an endometriosis lesion is an emerging yet underutilized method to study endometriosis. By comparison, tissue explants have been extensively utilized in tumor research, particularly in ovarian tumors. This technology is advantageous due to the ability to conserve tissue architecture, spatial organization, and the lesion microenvironment. These components can be visualized using imaging methods (105, 106). Additionally, explant cultures can be established rapidly with minimal tissue manipulation beyond dissection and culture, however, maintaining cell viability remains a challenge due to the absence of functional vasculature. Tissue explants offer a promising *ex vivo* model to recapitulate the fibrotic microenvironment and study disease mechanisms (Figure 2D). Recent advancements, such as those demonstrated by Vissers et al., show that cryopreserved endometriotic tissue fragments remain viable after thawing and during at least 3 days of culture, enabling an efficient platform for testing therapeutic agents like anti-fibrotics (107). This approach could bridge gaps in endometriosis modeling by preserving the complex interplay of stromal, epithelial, and immune components required for the establishment and persistence of the endometriotic lesion.

### 7.6 Microfluidic 3D systems

Microfluidic organ−/tissue-on-a-chip systems enable the investigation of cell organization and communication in dimensions that mimic the ones of the natural tissue environment next to the integration of perfusion and mechanical cues (108, 109). In organoid models, cellular differentiation processes lead to tissue structure, whereas in organ-on-a-chip systems, the structure is imposed by the system’s design. Chen et al. first used an organ-on-a-chip system to demonstrate the importance of the patient’s peritoneal physiology and microenvironment on stromal cell invasion of the mesothelial cell layer during lesion formation. The group utilized microfluidic channels with coverslips to embed the endometrial stromal cells and human peritoneal mesothelial cells from control and endometriosis individuals onto a glass slide, after which the barriers were removed to allow cell migration. The mesothelial cells isolated from endometriosis patients gradually lost their adhesion capability to each other and the substrate upon invasion of the stromal cells and underwent apoptosis when surrounded by stromal cells (110). As an endometrial model, Gnecco et al. co-cultured primary human endothelial cells and endometrial stromal cells in a microfluidic organ-on-chip model of endometrial perivascular stroma to study stromal decidualization (differentiation of specialized stromal fibroblasts under the influence of ovarian hormones) and endometrial vascular function in physiological conditions (111). More recently, Ahn et al. established a vascularized endometrium-on-a-chip with three distinct layers: epithelium, stroma and blood vessels that recapitulates endometrial vasculo-angiogenesis and respond to hormonal stimuli (112).

Whereas not yet explored in the context of endometriosis, microfluidic systems can combine classical 2D or 3D cell culture with organoids/spheroids, similar to the intestine-on-a-chip system (113) (Figure 2F). In endometriosis research, such a system would have particular strengths, as it would allow embedding of the endometrial glandular structure into an environment that closely mimics the stroma of an endometriotic lesion *in vivo*, for example, an extracellular matrix with myofibroblasts and profibrotic immune cells.

In addition to self-organization during differentiation and mechanical cues, bioprinting achieves 3D organization on a cellular level. 3D bioprinting implements the structured deposition of cells embedded in a biocompatible matrix onto a biocompatible scaffold or a 3D mold, resulting in a 3D tissue with the specific and exact allocation of cells, matrix, and biomolecules to mimic the *in vivo* tissue architecture and functionality (114–118). Wendel et al. established the first scaffold-free bio-fabricated *in vitro* model of endometriosis and the endometriotic microenvironment. They utilized 12Z spheroids and heterotypic spheroids also containing human endometrial stromal cells, which were bio-fabricated onto a three-dimensional tissue construct. The spheroids maintained the same growth pattern after bio-fabrication and presented an innovative tissue-like scaffold-free model of endometriosis. The team demonstrated the ability to culture lesions with multiple cell types and study their interactions in an endometriotic microenvironment (119) (Table 1).

**Table 1:** Comparison of experimental *in vitro*, *ex vivo* and engineered models used in endometriosis research.

Model type	Cell source	Key features	Strengths	Limitations	Research relevance	References
2D cell culture (cell lines/primary cells)	Immortalized epithelial cell lines (e.g., 12Z, 11Z, 16), stromal cell lines, primary cells from patients	Express key markers, allow transwell immune assays, ECM use for invasion study	Robust characteristics, immortalized, suitable for cell signaling and epigenetics, cell crosstalk	Loss of *in vivo* morphology, potential changes of epigenetic markers, primary cell harvest/viability limits	Study signaling, epigenetics, epithelial-stromal/immune interactions	Fan (75), Banu et al. (76), Zeitvogel et al. (77), Banu et al. (78), Colón-Caraballo et al. (79), Arosh et al. (80), Marquardt et al. (81), Woo et al. (82), Pampaloni et al. (84), Baker and Chen (85), Hickman et al. (86), Kapałczyńska et al. (87), Pollock et al. (88), Gordon et al. (90)
3D spheroid models	Aggregates of epithelial cells (immortalized/primary) or stromal/epithelial co-culture	Self-organization, gene expression more similar to 3D tissue, model peritoneal invasion	Recapitulate invasion, cell interplay	Spherical, not full lesion, stromal core instead of *in vivo* structure	Study invasion, migration, cell–cell interaction	Brueggmann et al. (83), Song et al. (93)
Organoids (healthy tissue)	Human/murine adult endometrial stem cells	Hollow gland structure, polarity, secrete factors, maintain hormone receptors	Mimic endometrium, patient-specific, hormone response	Not direct from lesions, may lack lesion-specific features, require specific growth factor cocktail, donor-dependent variation is benefit and limitation	Study morphogenesis, hormone, cycle responses	Turco et al. (94), Boretto et al. (95)
Organoids (epithelial + stromal)	Co-culture of endometrial epithelial and stromal cells	Self-organize, polarity, show hormone/function markers	Mimic epithelial-stromal interaction, cycle/inflammation changes	Internal architecture not same as in vivo	Model crosstalk, cycle, inflammation	Gnecco et al. (97), Teerawat et al. (98)
Organoids (endometriosis-derived)	Patient lesion-derived cells	Disease transcriptional/epigenetic profile, altered TGF-β, hormone resistance, invasion, stratified epithelium, form lesions in immunocompromized mice	Capture patient lesion complexity, hormone resistance, possible drug testing	Require patient-sourced material, patient−/lesion-specific variation is benefit and limitation	Best for actual lesion features, structure, drug testing	Boretto et al. (101), Zhang et al. (104)
Microfluidic 3D systems	Organ/tissue-on-a-chip, peritoneal cell and stromal cell co-cultures, perfused flow	Mimics mechanical cues, perfusion, invasion; enables vascular/decidualization study	Physiological mimic of peritoneum, flow, stroma-endothelium	Potential technical complexity in particular for control of oxygen pressure/mechanical cues	Promising for invasion/stroma/vascular modeling	Yan et al. (108), Chen et al. (110), Gnecco et al. (111), Ahn et al. (112)
3D bioprinting models	Scaffold-free, generation of 3D structures of cells/spheroids embedded into extracellular matrix	Control of 3D architecture, variation of extracellular matrix	Tissue-scale, multicellular, engineered complexity	Benefit over other models needs to be shown, lack of vascularization	Tissue architecture	Wendel et al. (119)
*Ex vivo* patient explants	Direct culture of patient lesion tissue	Maintains aged/fibrotic, native microenvironment	Models chronicity/fibrosis, captures original tissue	Scalability limits, viability, heterogeneity	Aging, fibrosis, studies of physiological micro-environment	Vissers et al. (107)

Each model type is described by its cell source, structural and functional characteristics, major strengths, and key limitations. Research relevance is highlighted to show the most appropriate applications, such as signaling and epigenetic studies (2D cell cultures), invasion and migration (spheroid models), hormone and cycle responses (healthy tissue-derived organoids), epithelial-stromal interactions (co-culture organoids), lesion-specific biology and drug testing (endometriosis-derived organoids), microenvironment and vascular dynamics (microfluidic systems), engineered lesion simulation (3D bioprinting), and studies of chronicity and fibrosis (patient explants). These models provide complementary insights but differ in how closely they replicate the *in vivo* lesion microenvironment and their scalability for experiments.

### 7.7 A critical evaluation of 3D systems for disease modeling

Organoids, microfluidic systems and 3D bioprinting provide the means to create tissue structures that recapitulate the histology of endometriosis lesions. These models sustain the migrating cell/stem cell self-organization along with lineage commitment, while maintaining communication between different compartments in a spatially defined manner. One should note though, that some models described above are endometrial and not endometriotic. However, their application for endometriosis research would be possible. Thus, the question arises as to which degree the recapitulation of lesion structure is sufficient to mimic the pathophysiology of the lesions.

Through reference to the first part of this review, we analyse the different elements still missing in the current systems that are critical for understanding the etiology and disease progression *in vivo,* and thus highly relevant for the search for novel therapeutic interventions. In our view, four aspects need to be addressed, which are (i) the type of lesion, (ii) the cellular and physicochemical microenvironment, (iii) the aging of lesions, and (iv) the etiology of the lesion.

#### 7.7.1 Type of lesion

Superficial lesions, deep infiltrating lesions and endometriomata strongly differ in the histological structure and gene expression patterns (120, 121). Still, for the 3D models summarized above, there is little information regarding the type of lesion they intend to replicate.

The key components of a lesion are endometrial epithelial cells, endometrial stromal cells, macrophages, and other immune cells (122). However, each component is present in different proportions in each lesion type. Single-cell RNA-seq data showed that in endometriomata epithelial cells are significantly depleted, while B cells and plasma cells are enriched.

In contrast, extra-ovarian endometriosis lesions have an increased presence of mast cells, myeloid cells, and T/NK cells. Both types of lesions contain fibroblasts, stromal cells, smooth muscle cells and endothelial cells (123). Most of the *in vitro* models only focus on the endometrial epithelial cells and stromal cells to model the peritoneal lesion. With progress in the complexity of the disease models, other cell types should be included in the model systems, specifically as they set endometriosis apart from the endometrium. Adjusting their proportions to mimic the different types of endometriosis lesions and their microenvironment will also be important. Recapitulating their functional characteristics imposes a further challenge.

In addition, endometriomata are often characterized by the presence of hemosiderin-laden macrophages and a fibrotic capsule, while deep infiltrating endometriosis may involve significant smooth muscle metaplasia. Currently, no model has attempted to incorporate these features. Recapitulation of mechanical and chemical features has been achieved in other contexts (see below).

In principle, the influence of a particular cell type on a model system can be examined using two different approaches. First, attempts could be made to isolate cells such as the hemosiderin-laden macrophages from patient lesions and assess their impact on the model upon incorporation. Additionally, *in vitro* differentiation from precursor cells could be an option. Second, a model lacking a cell type could be compared to the *in vivo* situation, and it could then be assumed that at least a subset of identified differences is due to the lack of the respective cell type. For the fibrotic capsule, one could consider using a decellularized endometrioma cyst as a scaffold. Similarly, incorporating the fluid of the chocolate cyst in an endometrioma model may be highly instructive. The fluid in the chocolate cysts consists of degraded blood and contributes to the chemical microenvironment. It has been shown that exposure to cyst fluid enhances reactive oxygen species in cells (124).

Bioprinting can be useful for the generation of models for deep infiltrating lesions. Organoids and the latest bioprinting technologies have successfully created structurally intact models with glandular and stromal compartments of endometriotic tissue. These models can be further improved by incorporating gland-like structures surrounded by stromal cells in smooth muscles.

#### 7.7.2 Microenvironment

The microenvironment of endometriotic lesions is critical in influencing disease progression. The epithelial glandular cells are exposed to various environmental cues, including immune cells, blood cells, and inflammatory and chemical mediators with the cyst fluid as one particular case. Furthermore, fibrotic changes increase stiffness. These interactions can significantly affect the behavior of the epithelial cells, such as their proliferation, invasion, and response to treatment. For example, macrophages and other immune cells create a pro-inflammatory environment that promotes lesion growth, fibrosis, and resistance to apoptosis (125). Cell-derived environmental cues such as cytokines can either be delivered by implementing the respective cell types as described above or by adding the isolated factors instead.

Importantly, the endometriotic lesions often exist in a hypoxic environment, which can influence cell behavior and gene expression. Hypoxic signals have been reported to activate epithelial-to-mesenchymal transition (EMT) in endometriosis, potentially contributing to smooth muscle metaplasia and fibrosis as seen in endometriosis lesions (126, 127). Researchers have developed 3D models that replicate this hypoxic condition by culturing cells in low-oxygen chambers or by using hypoxia-inducing agents (128, 129). These models have been used to study the effects of low oxygen on cell proliferation, invasion, and angiogenesis.

#### 7.7.3 Aging of the lesion

Endometriotic lesions can persist for years, potentially starting with menarche and continuing after menopause (130). The age of endometriotic lesions influences their cellular composition, tissue structure, and response to treatment. Older lesions tend to have more extensive fibrosis, thus more stiffness, and altered gene expression profiles due to epigenetic modifications (131). Therefore, it is important to consider the temporal component when developing 3D models. In the systems described above, the lesions are generated from established cell lines or stem cells using collagen as an extracellular matrix. Very clearly, these systems represent young lesions. Since organoid models inevitably can only capture short time frames over several days to a few weeks at best, patient-derived tissue explants provide access to aged lesions (107). However, protocols must be devised to characterize these explants with respect to age and to properly place results into context. Since epigenetic changes, such as DNA methylation and histone modifications, play a crucial role in the development and progression of endometriosis, analysis of these epigenetic modifications may also be part of characterizing *ex vivo* tissue explants. As a 3D model system, organoids have been treated with epigenetic modulators to investigate changes in gene expression and cellular behavior, thereby providing insights into the role of epigenetics in endometriosis (132). Placing such models into a matrix with increased stiffness may move them further toward an aged phenotype (133, 134).

#### 7.7.4 Etiology

As outlined in the first part of this review, one of the main challenges is the need for a better understanding of the disease etiology in relation to the various lesion types. Most *in vitro* endometriosis models, including spheroids and organoids, are used to study the attachment of endometrial cells onto the peritoneal or ovarian surface. In these models, retrograde menstruation and stem cell theories assuming an endometrial origin are the presumed etiologies of the disease. Coelomic metaplasia and embryonic rest theory have been less commonly studied. We are only aware of Matsuura et al., who described the metaplastic transformation of ovarian surface epithelial cells to form endometrial-like lumen structures upon exposure to oestradiol (59). This study also demonstrated that this etiology is readily accessible to investigation by model systems. For instance, models could be designed by co-culturing mesothelial cells with menstrual effluents, as previous studies have demonstrated that such exposure can induce mesothelial-to-mesenchymal transition (MMT) and morphological changes in these cells (135, 136). These models could further incorporate investigations into the effects of estrogen, either alone or in combination with effluent components, to better explore hormonal influences on metaplasia and MMT in endometriosis pathogenesis.

By comparison, designing models with Müllerian duct cells to study differentiation during embryogenesis is challenging since the starting material is already difficult to obtain, as Müllerian ducts are only present in fetuses or remnants that may be found in adults as a developmental anomaly. To overcome this limitation and model the embryonic origins of endometriosis, human pluripotent stem cells (hPSCs), including induced pluripotent stem cells (iPSCs), can be directed to form Müllerian duct-like cells. These cells have been shown to differentiate into endometrial epithelial and stromal cells, recapitulating key developmental stages of the Müllerian duct (137). Such hPSC-based models enable research into aberrant differentiation or ectopic lesion formation and provide insights into how misplaced Müllerian progenitors may contribute to endometriosis pathogenesis.

As an important final remark, at present, all models exclusively address the process of lesion formation. It should also be investigated under which conditions no lesion is formed, as in most women, retrograde menstruation does not lead to endometriosis.
